# Cu-doped SnO_2_/rGO nanocomposites for ultrasensitive H_2_S detection at low temperature

**DOI:** 10.1038/s41378-023-00517-z

**Published:** 2023-05-30

**Authors:** Tingting Chen, Jianhai Sun, Ning Xue, Wen Wang, Zongchang Luo, Qinqin Liang, Tianye Zhou, Hao Quan, Haoyuan Cai, Kangsong Tang, Kaisheng Jiang

**Affiliations:** 1 State Key Laboratory of Transducer Technology, Aerospace Information Research Institute, Chinese Academy of Sciences, 100194 Beijing, China; 2 School of Electronic, Electrical and Communication Engineering, University of Chinese Academy of Sciences, 100049 Beijing, China; 3 State Key Laboratory of Acoustics, Institute of Acoustics, Chinese Academy of Sciences, 100190 Beijing, China; 4 Guangxi Key Laboratory of Intelligent Control and Maintenance of Power Equipment, School of Electronic Engineering, Guangxi University, Nanning, 530004 Guangxi China; 5 Electric Power Research Institute of Guangxi Power Grid Co., Ltd., Nanning, 530013 Guangxi China

**Keywords:** Organic-inorganic nanostructures, Sensors

## Abstract

Hydrogen sulfide (H_2_S) detection remains a significant concern and the sensitivity, selectivity, and detection limit must be balanced at low temperatures. Herein, we utilized a facile solvothermal method to prepare Cu-doped SnO_2_/rGO nanocomposites that have emerged as promising candidate materials for H_2_S sensors. Characterization of the Cu-SnO_2_/rGO was carried out to determine its surface morphology, chemical composition, and crystal defects. The optimal sensor response for 10 ppm H_2_S was ~1415.7 at 120 °C, which was over 320 times higher than that seen for pristine SnO_2_ CQDs (*R*_a_/*R*_g_ = 4.4) at 280 °C. Moreover, the sensor material exhibited excellent selectivity, a superior linear working range (*R*^2^ = 0.991, 1–150 ppm), a fast response time (31 s to 2 ppm), and ppb-level H_2_S detection (*R*_a_/*R*_g_ = 1.26 to 50 ppb) at 120 °C. In addition, the sensor maintained a high performance even at extremely high humidity (90%) and showed outstanding long-term stability. These superb H_2_S sensing properties were attributed to catalytic sensitization by the Cu dopant and a synergistic effect of the Cu-SnO_2_ and rGO, which offered abundant active sites for O_2_ and H_2_S absorption and accelerated the transfer of electrons/holes.

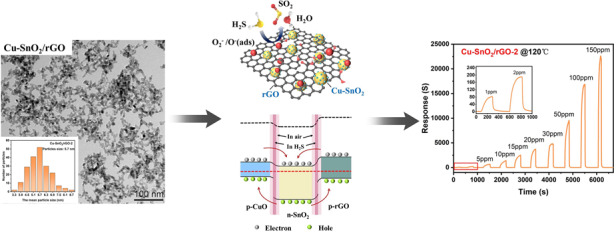

## Introduction

Hydrogen sulfide (H_2_S) is a toxic pollutant gas that degrades the air quality and has negative effects on human health even at low concentrations (10 ppm)^1,2^. On the other hand, H_2_S at the ppb level is also an essential indicator used for diagnoses of diseases, such as diabetes, liver cirrhosis, and asthma^3–5^. Therefore, the safety and health of human beings requires highly sensitive monitoring of low H_2_S concentrations.

Colloidal quantum dots (CQDs) are semiconductor nanocrystals with physical dimensions below their Bohr radii, and they are usually synthesized and processed in solution^6^. They present numerous gas-sensing advantages, such as large specific surface areas, porous film structures, and easy integration on virtually any substrate^7,8^. Metal oxide CQDs are commonly used in the design and fabrication of gas sensors. Xu et al. first synthesized SnO_2_ QDs for the detection of ethanol using a mixed solvent system composed of oleylamine and oleic acid, but the gas-sensing performance was inevitably hindered by the organics covering the surface^9^. Liu et al. proposed an innovative solution to this problem by utilizing inorganic salts for subsequent surface ligand treatments^8^. This strategy was useful in dealing with relatively large-volume ceramic substrates, yet it was not ideal for micro heating plates with low power consumption. Although various SnO_2_ QD/QW-based sensors have been synthesized for H_2_S, NH_3_, and NO_2_ detection based on this approach, they still generally suffer from many defects that hinder practical application, such as a strong dependence on ambient humidity, low sensitivity, and a high limit of detection (LOD)^10,11^.

Copper (Cu) is an excellent and sensitive catalyst, and it has recently been dispersed on the surfaces of SnO_2_ films to form islands or continuous layers exhibiting selective adsorption of H_2_S^12^. It was found that Cu doping resulted in Cu^2+^ occupation of Sn^4+^ sites and generated a large number of oxygen vacancies to maintain the charge neutrality, which resulted in enhanced gas-sensing performance of the oxide semiconductor sensors. Additionally, CuO reacts with H_2_S gas to improve the selectivity of the sensor^13^. However, the Cu-doped SnO_2_ generally operates at high temperatures (>180 °C), which increases the power consumption of the sensor^12–14^.

Reduced graphene oxide (rGO)-based layered nanomaterials are two-dimensional carbon materials that have proven to be excellent candidates for decorating MOS gas-sensing materials and enabling them to work at low temperatures^15,16^. This is because of its high surface area to volume ratio, high charge carrier mobility (200,000 cm^2^/V s at room temperature), active defect sites, and detectable single molecule adsorption/desorption^17^. Furthermore, chemical functionalization with metal and metal oxide nanoparticles allows facile detection of many analytes at low concentrations^18–20^. For example, Cui et al. synthesized In-doped SnO_2_/rGO composites via a one-pot hydrothermal method, and they exhibited high selectivities and gas responses at room temperature for NO_2_, with LODs as low as 0.3 ppm^20^. The use of Cu and rGO for SnO_2_ sensing, therefore, shows promise for fabricating H_2_S sensors with high sensitivities, low detection limits, and short response/recovery times. To the best of our knowledge, there have been no reports on the utilization of Cu-doped SnO_2_/rGO for H_2_S sensing.

Herein, the solvothermal method was employed in conjunction with high-temperature annealing to synthesize sensing materials utilizing oleic acid and oleylamine as solvents and surfactants, as well as to investigate their use in H_2_S sensing. The as-synthesized Cu-SnO_2_/rGO had grain sizes of nearly 5.7 nm and surfaces rich in adsorbed oxygen and oxygen vacancies, combined with large BET surface areas and pore sizes. Hence, it exhibited remarkably higher sensitivity (156.5 ppm^−1^) and much lower detection limits (50 ppb) for H_2_S detection than state-of-the-art sensors. A thorough study of the gas-sensing mechanism indicated that the dramatic enhancement in H_2_S sensing performance was mainly dependent on the synergistic effect of the doped Cu and rGO with SnO_2_. This work provides a new perspective for the study of high-performance H_2_S gas sensor fabrication.

## Results and discussion

### Characterization

**Fig. 1:**
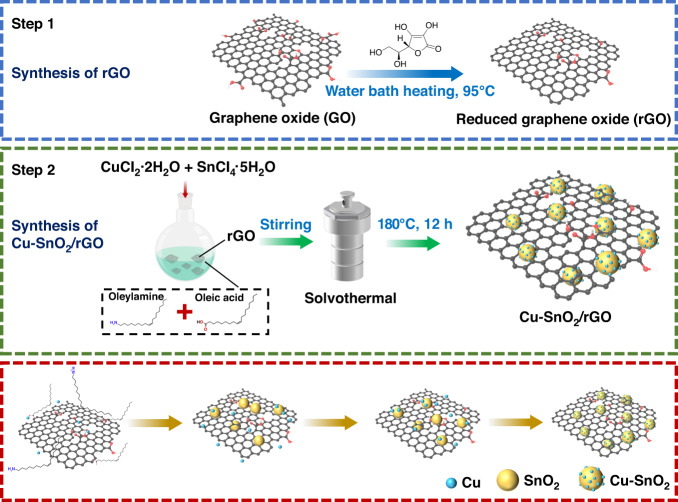
Experimental set-up for the nanocomposites. Schematic illustration for the synthesis of the Cu-SnO_2_/rGO nanocomposite via the solvothermal method

A schematic diagram for the synthesis of Cu-SnO_2_/rGO is illustrated in Fig. 1. First, the weakly reducing L-ascorbic acid was utilized to reduce the GO. Second, a mixed solvent consisting of oleylamine and oleic acid was employed as a surfactant to control the growth of SnO_2_ nanoparticles, and Cu^2+^ and the as-synthesized rGO were incorporated as dopants and reacted together. Finally, the Cu-SnO_2_/rGO nanocomposites were prepared via high-temperature annealing (400 °C).

**Fig. 2:**
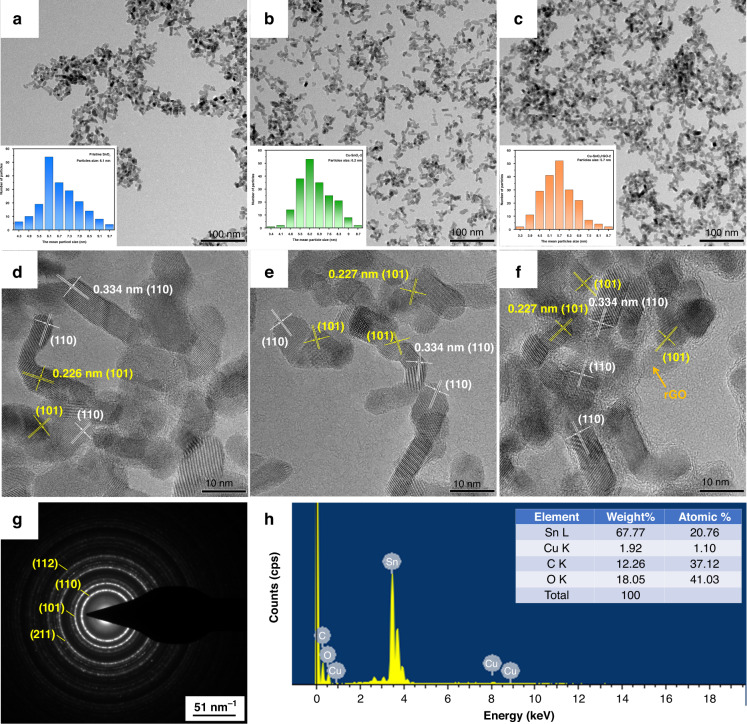
TEM characterization of nanocomposites. TEM and HRTEM images of the pristine SnO_2_ CQDs (**a**, **d**), Cu-SnO_2_-2 (**b**, **e**), and Cu-SnO_2_/rGO-2 (**c**, **f**). The insets in (**a**–**c**) show the particle sizes estimated from 200 samples in the TEM images. **g** SAED pattern and **h** EDS spectrum of the as-synthesized Cu-SnO_2_/rGO-2 nanocomposite

In Figs. 2a–c and S1, the TEM and SEM images illustrate the morphologies of the pristine SnO_2_ CQDs, Cu-SnO_2_-2, and Cu-SnO_2_/rGO-2 (Fig. 2a–c), all of which comprised randomly shaped nanoparticles. The synthetic rGO had a lamellar structure with folds. The nanocrystalline sizes of these three samples were obtained by measuring 200 particles with the software Nano Measure. Thus, their average diameters were estimated to be 6.1, 6.2, and 5.7 nm, respectively. The smaller size of the Cu-SnO_2_/rGO-2 particles was attributed to the planar hydrophilic edges of the rGO acting as surface ligands bound to Sn^4+^, which limited the nucleation and growth of the SnO_2_ nanocrystals^21^. It should be noted that the Cu-SnO_2_ grain sizes were slightly expanded compared to those of the pristine SnO_2_ CQDs because the Cu 2p ionic radius (0.87 Å) is larger than that of Sn 4b (0.83 Å) and the Sn^4+^ ions were replaced by Cu^2+ ^^22^. The HRTEM images of the pristine SnO_2_ CQDs, Cu-SnO_2_-2, and Cu-SnO_2_/rGO-2 exhibited high degrees of crystallinity, as shown in Fig. 2d–f. The separation distances of the (110) and (101) planes were estimated as ~0.334 and 0.266 nm, respectively. The four well-defined diffraction rings for the selected area electron diffraction (SAED) patterns shown in Fig. 2g corresponded to the (110), (101), (211), and (112) planes, confirming the tetragonal rutile structure of SnO_2_^23^. The EDS image for Cu-SnO_2_/rGO revealed that the nanocomposites were doped with copper at an atomic ratio of ~1%, as shown in Fig. 2h.

**Fig. 3:**
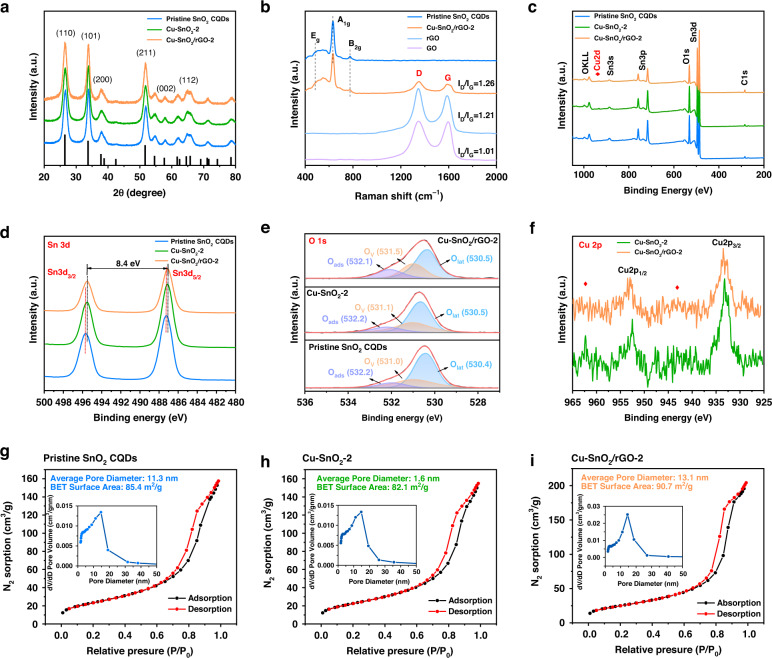
XRD, Raman, XPS and N_2_ sorption−isotherms characterization of nanocomposites. **a** XRD patterns of the pristine SnO_2_ CQDs, Cu-SnO_2_-2, and Cu-SnO_2_/rGO-2; the JCPDS No. 41-1445 diffraction pattern is presented at the bottom. **b** Raman spectra of the pristine GO, rGO, SnO_2_ CQDs, and Cu-SnO_2_/rGO-2 nanocomposites. **c** X-ray photoelectron spectroscopy (XPS) survey spectra and high-resolution XPS spectra of the pristine SnO_2_ CQDs, Cu-SnO_2_-2, and Cu-SnO_2_/rGO-2: **d** Sn 3d, **e** O 1s, and **f** Cu 2p binding energies. N_2_ sorption−isotherms and pore width distributions at 77 K for the (**g**) pristine SnO_2_ CQDs, (**h**) Cu-SnO_2_-2, and (**i**) Cu-SnO_2_/rGO-2

The three strongest peaks contained in the XRD patterns of the three samples in Fig. 3a were situated at 26.6°, 34.0°, and 51.8° (2*θ*), and these corresponded to the (110), (101), and (211) crystallographic facets of the tetragonal rutile SnO_2_ structure (JCPDS No. 41-1445), respectively^24^. No additional features associated with CuO/Cu_2_O were observed. Nevertheless, as the amount of Cu doping was increased, the positions of the (100) and (101) peaks for the samples shifted toward lower 2*θ* values, as shown in Fig. S2a. This indicated that the Cu^2+^ replaced some of the Sn^4+^ in the SnO_2_ lattice to form a solid solution phase, in agreement with the XPS results^25^. No rGO diffraction peaks were observed for the Cu-SnO_2_/rGO nanocomposites (Fig. S2b), probably owing to the low rGO doping level and relatively weak peak intensity indicating that the rGO could not change the lattice structure, consistent with the HRTEM and SAED results. In addition, the characteristic peak intensity for SnO_2_ gradually increases with increasing rGO doping, which indicated a continuous increase in the SnO_2_ crystallinity. No (002) Bragg peak was observed for rGO.

Raman spectroscopy was utilized to illustrate the reduction of GO and the synthesis of Cu-SnO_2_/rGO. As shown in Fig. 3b, the Raman peaks for the pristine SnO_2_ CQDs and Cu-SnO_2_/rGO-2 at 474, 632, and 778 cm^−1^ corresponded to the E_g_, A_1g_, and B_2g_ vibrational modes of tetragonal rutile SnO_2_, respectively. GO, rGO, and Cu-SnO_2_/rGO-2 showed the two intense and characteristic graphene peaks situated at 1351 and 1589 cm^−1^ corresponding to the D and G bands. The vibrations of the sp^2^-bonded carbon atoms are represented by the G-band, whereas the structural defects are associated with the D-band^26^. The intensity ratios of the D to G bands (*I*_D_/*I*_G_) are related to the number of functional groups in the rGO, and these were 1.01, 1.21, and 1.26 for GO, rGO, and Cu-SnO_2_/rGO-2, respectively. A higher value indicated partial modification of the surface oxygen-containing functional groups and the formation of heterojunctions between rGO and Cu-SnO_2_^27,28^.

The XPS survey spectrum for Cu-SnO_2_/rGO-2 indicated Sn, Cu, O, and C peaks and no impurity peaks, as shown in Fig. 3c. The C 1s peak at 284.8 eV was attributed to the surface carbon of the XPS instrument. The spectra for the pristine SnO_2_ CQDs confirmed the presence of Sn, O, and C only, which indicated that Cu was successfully doped in Cu-SnO_2_/rGO-2. The peaks at 495.67 and 487.27 eV in Fig. 3d corresponded to the Sn 3d_3/2_ and Sn 3d_5/2_ binding energies of Sn^4+^, indicating the formation of SnO_2_^29^. The excellent symmetric shapes of these peaks excluded the presence of metallic tin. The disparity in binding energy (0.16 eV) was probably due to the Sn-O interactions resulting after doping with Cu. The high-resolution O 1s spectrum showed peaks for the three samples (Fig. 3e) that were split into three Gaussian peaks and attributed to the three chemical states of O. The O 1s peaks adjacent to 530.5 eV were attributed to the O^2−^ in the SnO_2_ crystal lattice, which is designated lattice oxygen (O_lat_)^30^. The peak near 531.0 eV was for oxygen vacancy (O_v_), which is attributed to the oxygen-related vacancies in the SnO_2_ crystallographic structure^31^. The peak at ~532.1 eV was for absorbed oxygen (O_ads_), the oxygen species(s) adsorbed by the materials^32^. These three oxygen species are of great importance for gas sensing and will be investigated separately in the Sensing Mechanism section. For Cu-SnO_2_-2 and Cu-SnO_2_/rGO-2, the high-resolution Cu 2p spectrum showed four peaks (Fig. 3f). The peaks at 952.5 and 933.2 eV indicated the Cu 2p_1/2_ and Cu 2p_3/2_ binding energies, respectively, which confirmed the presence of Cu^2+^ and Cu^+^ ions. The two Cu 2p satellite peaks near 962.3 and 942.9 eV corresponded to the CuO phase^14^.

**Fig. 4:**
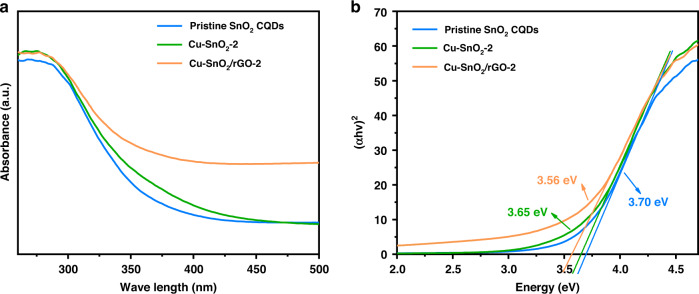
UV−vis diffuse reflectance spectra and *h*ν vs. (α*hν*)^2^ curves. **a** UV–vis diffuse reflectance spectra and **b** Kubelka–Munk function curves plotted against the energy of light absorbed by the pristine SnO_2_ CQDs, Cu-SnO_2_-2, and Cu-SnO_2_/rGO-2. The bandgaps were 3.70, 3.65, and 3.56 eV, respectively

The N_2_ sorption−isotherms of the pristine SnO_2_ CQDs, Cu-SnO_2_-2, and Cu-SnO_2_/rGO-2 are presented in Fig. 3g–i. The distinct hysteresis loops of the three samples indicated the presence of mesopores^33^. Doping with rGO endowed the Cu-SnO_2_/rGO-2 surface with a larger average pore size (13.1 nm) and increased Brunauer–Emmett–Teller (BET) surface area (90.7 m^2^ g^−1^) than the pristine SnO_2_ CQDs (11.3 nm; 85.4 m^2^ g^−1^) and Cu-SnO_2_-2 (11.6 nm; 82.1 m^2^ g^−1^). The larger average pore size facilitated the transport of H_2_S molecules between the ex- and internal regions to enable swift response/recovery even at low temperatures; moreover, the higher BET surface area provided more gas absorption and active sites. Noticeably, the Cu-doped SnO_2_ showed a slightly lower specific surface area than the pristine SnO_2_ CQDs, probably because the originally doped Cu occupied some channels of the SnO_2_. Furthermore, based on UV‒vis adsorption spectra, we obtained band gaps of 3.70, 3.65, and 3.56 eV for the pristine SnO_2_ CQDs, Cu-SnO_2_-2 and Cu-SnO_2_/rGO-2, respectively, after transformation, as shown in Fig. 4. The narrower bandgap indicated that the electrons in Cu-SnO_2_/rGO-2 transitioned more conveniently from the valence band to the conduction band, which enabled gas sensing and required lower activation energies for chemical reactions^34^.

### Gas-sensing performance

**Fig. 5:**
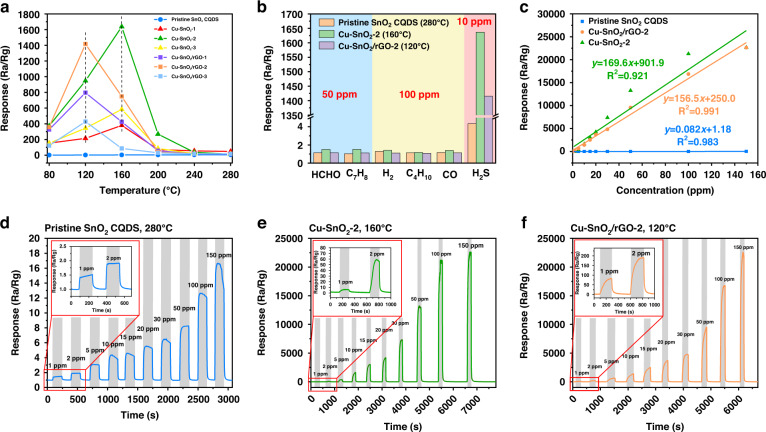
Gas sensing properties of the Cu-SnO_2_/rGO based sensors. **a** Gas-sensing response versus operating temperature for detection of 10 ppm H_2_S; **b** selectivities toward HCHO (50 ppm), toluene (50 ppm), H_2_ (100 ppm), n-butane (100 ppm), and CO (100 ppm); **c**–**e** dynamic responses of sensors to various concentrations of H_2_S; **f** linear fitting of the sensor responses to various concentrations of H_2_S

The sensing capabilities of the pristine SnO_2_ CQDs, Cu-SnO_2_, and Cu-SnO_2_/rGO were systematically evaluated. The real-time resistance was monitored to identify the optimal operating temperature by exposing the SnO_2_-based sensors doped with different concentrations of Cu and rGO to 10 ppm H_2_S at different temperatures, as shown in Fig. 5a. The sensing response (*R*_a_/*R*_g_) to 10 ppm H_2_S of the pristine SnO_2_ CQDs exhibited a gradual rise as the temperature increased, and the highest response of 4.4 was attained at 280 °C. With various Cu doping amounts, the response values all peaked at the same temperature (160 °C), and they reached the highest level of 1636.8 for Cu-SnO_2_-2. Furthermore, the rGO dopant reduced the operating temperature down to 120 °C, and there was a peak in the sensing response of 1415.7 for Cu-SnO_2_/rGO-2. This was attributed to formation of a p-n heterojunction by the rGO and Cu-SnO_2_, which reduced the activation energy required for the chemical reaction between the semiconductor and the gas molecules. In addition, the underlying mechanism for the reduced operating temperature of the Cu-SnO_2_/rGO-based sensors compared to the pristine SnO_2_ CQDs and Cu-SnO_2_ can be explained in two ways. First, the rGO exhibited a large specific surface area and a high material submobility, which increased the number of active sites and provided a greater variety of surface adsorbed oxygen species. As shown in Table S1, the contents of O_ads_ in these three materials decreased in the order Cu-SnO_2_/rGO-2 (20.0%), pristine SnO_2_ CQDs (10.5%) and Cu-SnO_2_-2 (13.3%), indicating that doping with rGO activated and dissociated O_2_ from the ambient air and increased the content of O_ads_. The increased O_ads_ composition meant that more surface chemisorbed oxygen species were involved in oxidation‒reduction reactions, which reduced the activation energy for the reaction between the gas and adsorbed oxygen. Second, the narrower bandgap indicated that the electrons in Cu-SnO_2_/rGO-2 transitioned more readily from the valence band to the conduction band and lowered the activation energy required for the chemical reactions.

Hereafter, 280 °C, 160 °C, and 120 °C were chosen as the optimum operating temperatures for evaluating the H_2_S sensing properties of the pristine SnO_2_ CQDs, Cu-SnO_2_-2, and Cu-SnO_2_/rGO-2, respectively. The cross-responses to different gases have been important problems for MOS sensors. To assess the gas selectivity of the pristine SnO_2_ CQDs, Cu-SnO_2_-2, and Cu-SnO_2_/rGO-2, these sensors were treated at their operating temperatures with 50 ppm HCHO and C_7_H_8_ (toluene) and 100 ppm H_2_, C_4_H_10_ (n-butane), and CO. As shown in Fig. 5b, the responses of these sensors to the above gases were all less than 2, much lower than the responses to H_2_S; however, the selectivities of the Cu-SnO_2_-2 and Cu-SnO_2_/rGO-2 were significantly higher than that of the pristine SnO_2_ CQDs. The dynamic response-recovery transients for the pristine SnO_2_ CQDs (in blue), Cu-SnO_2_-2 (in green), and Cu-SnO_2_/rGO-2 (in orange) after H_2_S exposure/release cycles with different concentrations (1, 2, 5, 10, 15, 20, 30, 50, 100, and 150 ppm) are shown in Fig. 5c–e. The responses of the sensors rose sharply with increasing H_2_S concentration. The Cu-SnO_2_/rGO-2 sensor, for which the slope of the linear fit was ~156.5 ppm^−1^, showed a sensitivity enhanced by over 1900 times in comparison with the pristine SnO_2_ CQDs (Fig. 5f). Moreover, the Cu-SnO_2_/rGO-2 sensor featured a linear response (*R*^2^ = 0.991) compared with Cu-SnO_2_-2 (*R*^2^ = 0.921), which tended to become saturated at relatively large concentrations (>50 ppm). In summary, the Cu-SnO_2_/rGO-based sensor presented a better sensing performance than the pristine SnO_2_ CQDs and Cu-SnO_2_-2 in terms of operating temperature, linearity, and selectivity.

**Fig. 6:**
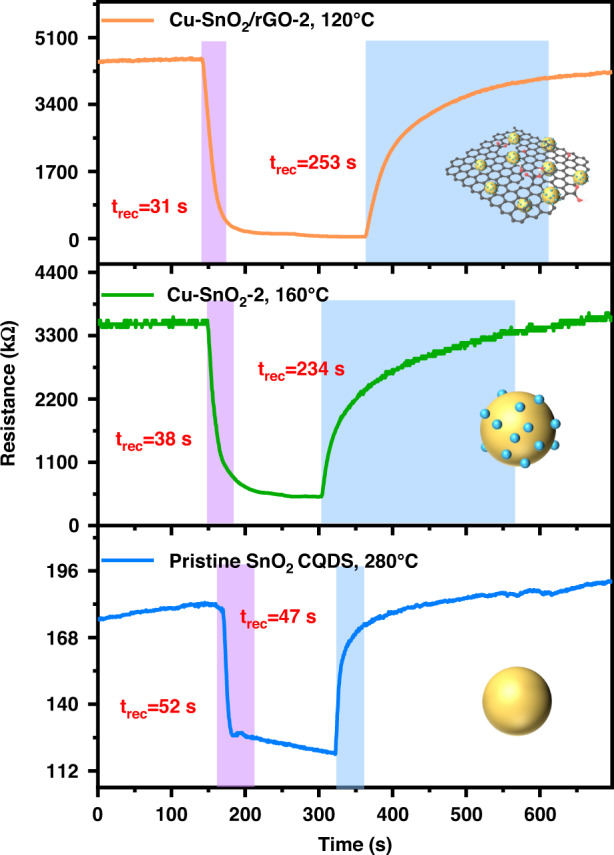
Response/recovery curves of the sensors. Response/recovery curves for the sensors subjected to 2 ppm H_2_S at their optimal working temperatures

Figure 6 presents the response/recovery curves of the three sensors exposed to 2 ppm H_2_S. The Cu-SnO_2_/rGO-based sensor worked at the lowest temperature and exhibited the shortest *t*_res_ (31 s), which was attributed to the larger specific surface area and the Cu-SnO_2_ and rGO heterojunction that reduced the activation energy for gas sensing and accelerated the reaction between H_2_S and the chemisorbed oxygen. In addition, the baseline resistance of Cu-SnO_2_/rGO-2 (~4.5 MΩ) was higher than those of the Cu-SnO_2_-2 (~3.5 MΩ) and pristine SnO_2_ CQDs (~0.2 MΩ). This may be due to the ternary heterojunctions that promoted the adsorption and decomposition of O_2_, which formed a higher concentration of chemisorbed oxygen on the surface and resulted in an increase in the thickness of the electric depletion layer.

**Fig. 7:**
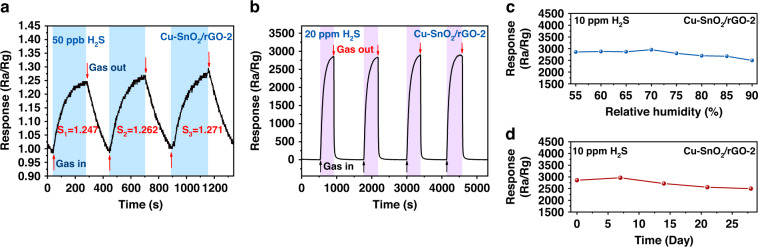
The sensing performance of the Cu-SnO_2_/rGO-2 based sensor. **a** three-cycle repeat detection of 50 ppb H_2_S, **b** four-cycle repeated detection, **c** sensing response curves under different relative humidities, and **d** long-term stability

Figure 7a illustrates that the sensor based on Cu-SnO_2_/rGO-2 attained an average of 1.26 for three sequential responses to 50 ppb H_2_S. Hence, we concluded that the LOD of this sensor was less than 50 ppb. As shown in Fig. 7b, the response of the sensor to 20 ppm H_2_S presented similar transients, and all of the resistance values recovered to the initial value for the four consecutive cycles, confirming the outstanding repeatability. Figure 7c displays the behavior of the Cu-SnO_2_/rGO-2 sensor as the relative humidity was varied from 55 to 90%. The responses of the sensor differed slightly as the ambient humidity increased, which indicated that it was only minimally affected by the humidity. In addition, the sensitivity of the Cu-SnO_2_/rGO-2 sensor was almost constant for 28 days, as demonstrated in Fig. 7d, and this indicated its good long-term stability. In Table S2, we have summarized the performance of the Cu-SnO_2_/rGO sensor and compared it with those of other SnO_2_-based H_2_S sensors reported in the recent literature for the sake of comparison. The Cu-SnO_2_/rGO-2 sensor prepared in this work showed high sensitivity, a low detection limit, and fast recovery at relatively low operating temperatures, which indicated that the prepared Cu-SnO_2_/rGO-2 sensor has broad development prospects and potential for use in H_2_S detection.

### Sensing mechanism

**Fig. 8:**
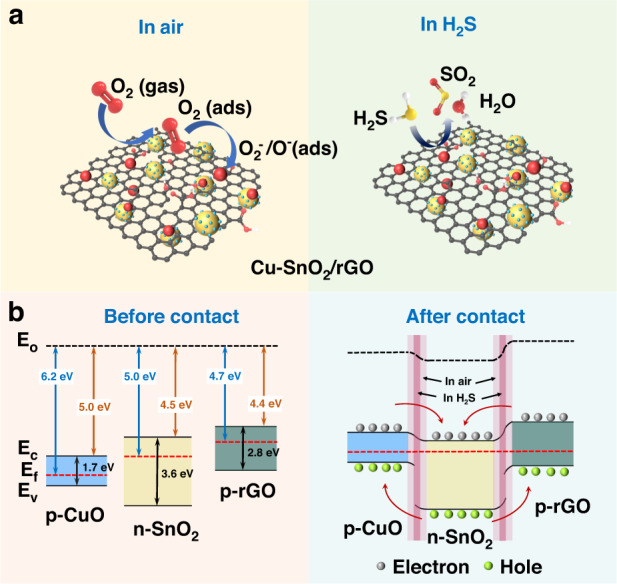
Gas sensing mechanism of the Cu-SnO_2_/rGO based sensor. **a** Schematic diagram of the gas-sensing mechanism for the sensor based on Cu-SnO_2_/rGO. **b** Energy band structure for the p-n-p heterojunction of Cu-SnO_2_/rGO. The band structure data in (**b**) were determined from the literature^40,41^

The sensing mechanism of the SnO_2_ (an n-type MOS) sensor involved gas adsorption and surface-related redox reactions^35^. A schematic of the gas-sensing mechanism is provided in Fig. 8a. For the Cu-SnO_2_/rGO synthesized in this work, oxygen molecules from the ambient atmosphere were adsorbed on the surface of the material and converted to oxygen anions by trapping electrons from the conduction band. Under the test conditions (120 °C), the surface oxygen species were primarily O^2−^ and O^−^^36^. The loss of electrons caused the formation of an electron depletion layer in the surface region, while a potential barrier was built between the adjacent grains; this impeded the flow of electrons at the grain boundaries, which manifested itself macroscopically as an increased resistance. When the sensor was exposed to H_2_S, oxygen ions reacted with H_2_S and delivered electrons to the Cu-SnO_2_/rGO surface. As a result, the electron depletion layer narrowed, the barrier between the adjacent grains was reduced, and the resistance decreased. The exact reactions are shown in ref. ^37^.

The sensor based on Cu-SnO_2_/rGO operated at the lowest temperature and showed higher sensitivity than the pristine SnO_2_ CQDs and Cu-SnO_2_ for the following reasons. First, Cu-SnO_2_/rGO formed a special p-n-p ternary heterostructure. The energy band structure is shown in Fig. 8b. Under an ambient atmosphere, electrons were transferred from SnO_2_ to the rGO and CuO, whereas holes were transferred in the opposite direction until the Fermi energy level reaches equilibrium. The electron depletion layer at the heterojunction interface was hence wider than that of pristine SnO_2_, corresponding to an increase in the baseline resistance. H_2_S reacted with the surface negative oxygen species, and electrons entered the conduction band of SnO_2_. Additionally, some electrons also entered the conduction band of rGO and CuO, which was manifested at the macroscopic level as an enhanced conductivity. In addition, the heterojunctions also contributed to the catalytic activity by providing more adsorption reaction sites^38^. Second, according to the three oxygen species occupancy ratios derived from the O 1s XPS data (Table S1), the Cu-SnO_2_/rGO surface had more adsorbed oxygen and oxygen vacancies. The abundant chemisorbed oxygen promoted adsorption and reaction of the reduced gas; the oxygen vacancies contributed to the increasing charge density near the valence and conduction bands, which narrowed the bandgap of SnO_2_ (Fig. 4) and facilitated adsorption and activation of the target gas. Moreover, the mesoporous structure provided effective diffusion channels for the gases, and the larger BET surface area provided more active sites for foreign oxygen molecules, which enabled penetration of the gaseous molecules and interactions with the interior grains. Finally, the Cu-SnO_2_/rGO grain size (5.7 nm) was the smallest and close to 2*L*_D_ (3 nm at 120 °C), which maximized the effect of varying the electron depletion layer thickness on the overall resistance.

## Conclusions

In summary, a Cu-doped SnO_2_/rGO-based H_2_S gas sensor was successfully synthesized via the solvothermal method. Compared to the pristine SnO_2_ CQDs and Cu-SnO_2_, the gas-sensing performance of the Cu-SnO_2_/rGO sensor was remarkably improved, with an ultrahigh sensitivity (156.6 ppm^−1^), an ultralow detection limit of 50 ppb (*R*_a_/*R*_g_ = 1.26), and a rapid response time (31 s, 2 ppm). In addition, the sensor operated effectively at high humidity (90%). These excellent H_2_S sensing properties were attributed to the synergistic effect of Cu and rGO with the SnO_2_: the smaller grain sizes, larger specific surface area, unique p-n-p heterostructure, increased oxygen vacancies, and narrower band gap structure increased the sensitivity of the sensor, and the larger pore size provided shorter response/recovery times for the sensor. Thus, these Cu-SnO_2_/rGO ternary nanocomposite sensors are promising candidates for fast, highly sensitive, and low-concentration detection of H_2_S. Additionally, we found that high-temperature annealing effectively reduced the organic coverage on the surface of SnO_2_ CQDs, which improved the gas-sensitive performance and reduced the influence of ambient humidity. We will study the mechanism of high-temperature annealing in future work.

## Materials and methods

### Chemical reagents

Graphene oxide (GO, >99%), tin (IV) chloride pentahydrate (SnCl_4_·5H_2_O, 99.0%), copper chloride dihydrate (CuCl_2_·2H_2_O), and oleylamine (C18, 80–90%) were obtained from Shanghai Macklin Biochemical Co., Ltd. Ascorbic acid (AA, ≥99.7%) and oleic acid (OA) were purchased from Shanghai Sinopharm Chemical Reagent Co., Ltd. All reagents were used in the experiments without further purification.

### Synthesis of rGO

GO was reduced by utilizing the green agent AA in a 95 °C water bath. In a typical procedure, GO (25 mg) was dispersed in deionized water (25 ml) and sonicated for 1 h to prepare a homogeneous GO dispersion (1 mg/ml). AA (250 mg) was then added to the GO dispersion and maintained at 95 °C in a constant temperature water bath for 24 h. After cooling to room temperature, the dispersion was rinsed 2–4 times with ethanol and deionized water to remove impurities. Finally, the rGO was dried in an oven at 75 °C overnight to obtain the rGO solid powder for characterization.

### Synthesis of Cu-SnO_2_/rGO nanocomposites

We synthesized the SnO_2_ CQDs via a slightly modified version of the solvothermal process reported by Xu et al.^9^. In a typical process, SnCl_4_ (1.2 mmol) and CuCl_2_ (0.6 mmol) were distributed in oleic acid (20 ml) and oleylamine (2.5 ml) by sonication for 10 min, followed by vigorous stirring at 60 °C to form a transparent solution. Subsequently, a rGO ethanol dispersion (3.6 ml, 1 mg/l) and ethanol (6.4 ml) were added in turn and stirred to achieve a hyaline. The solution was then transferred to a 50 ml Teflon-lined autoclave and maintained at 180 °C for 12 h. After natural cooling, the solution was washed with ethanol and hexane several times and then dried at 75 °C overnight. The as-synthesized powders were then calcined in a muffle furnace at 400 °C for 2 h with a 10 °C min^−1^ heating rate. The total molar ratio of SnCl_4_ to CuCl_2_ was kept constant, while varying the molar ratio of Cu^2+^ to Sn^4+^ yielded pristine SnO_2_ CQDs, Cu-SnO_2_-1 (1:4), Cu-SnO_2_-2 (1:2), and Cu-SnO_2_-3 (1:1). A series of Cu-SnO_2_/rGO nanocomposites were obtained with the indicated volumes of the rGO ethanol dispersions; Cu-SnO_2_/rGO-1 (1.8 ml), Cu-SnO_2_/rGO-2 (3.6 ml) and Cu-SnO_2_/rGO-3 (5.4 ml). A schematic diagram for the synthesis of Cu-SnO_2_/rGO is provided in Fig. 1.

### Characterization

The sizes and morphologies of the products were obtained by transmission electron microscopy and high-resolution transmission electron microscopy (TEM and HRTEM, FEI Tecnai G2 F30) with an energy-dispersive X-ray spectrometer (EDS, Xplore) operating at an accelerating voltage of 300 kV. The phase purities of the nanocrystals were determined with powder X-ray diffraction (XRD, Bruker D8) operating at 40 kV and 40 mA with Cu Kα irradiation (*λ* = 1.5406 Å). Scans were taken with a 2*θ* range of 20°–80° and step sizes of 6° min^−1^. Raman spectroscopy was performed with a HORIBA Scientific LabRAM HR Evolution instrument with the 514 nm line of an Ar^+^-ion laser. The surface compositions and bonding states of the nanocrystals were obtained by X-ray photoelectron spectroscopy (XPS, Thermo Scientific Escalab 250Xi) with an aluminum source; all binding energies were referenced to the C 1s peak at 284.8 eV for surface carbon. The specific surface areas and porosities of the as-synthesized samples were determined from nitrogen adsorption-desorption isotherms (Micromeritics TriStar III 3020) generated at 77 K. Ultraviolet–visible (UV‒vis) absorption spectra were measured with a Perkin–Elmer Model Lambda 950 UV–vis/NIR spectrophotometer.

### Gas-sensing measurements

The gas-sensing method was described in detail in our previous work^39^. Briefly, the as-synthesized samples were first well ground with agate and then mixed with ethanol to form a 15 mg/ml suspension. This was then coated on the micro thermal plate and dried at 60 °C for 2 h. For strong adsorption of the H_2_S, the purity of the gas was calculated with the stationary-state gas distribution method in this work. The desired concentrations of H_2_S (*C*) were obtained by diluting the standard H_2_S gas (100 ppm) with air as the background gas and calculated as *C* = *V*_s_ × *C*_s_/*V*, where *V*_s_ is the volume of standard gas that was injected into the chamber, *C*_s_ is the concentration of the standard gas (100 ppm standard gas mixed with clean air), and *V* is the volume of the sealed chamber (1 l). All measurements were carried out at ~55% RH and 25 °C, except for those determining the effect of humidity.

The sensor response *S* was calculated as *S* = *R*_a_/*R*_g_, where *R*_g_ and *R*_a_ are the resistance in the target gas and air, respectively. The response and recovery times (*t*_res_ and *t*_rec_) were defined as the time for the sensor to reach 90% of the total change in resistance. Moreover, the sensitivity of the sensor was expressed as the change in the measured response signal per ppm unit, i.e., the slope of the linearly fitted response line after calibration.

## Supplementary information

Supporting Information - Revised
