# Supplementary material for: Cu-doped SnO2/rGO nanocomposites for ultrasensitive H2S detection at low temperature
Source: Microsyst Nanoeng. 2023 May 30;9:69. doi: 10.1038/s41378-023-00517-z (PMC10227056; doi:10.1038/s41378-023-00517-z)
Supplement: Supplementary file 1 — Supporting Information - Revised [file 41378_2023_517_MOESM1_ESM.docx]

**Supporting Information**

**Cu-doped SnO_2_/rGO nanocomposites for ultrasensitive H_2_S detection under low temperature**

Tingting Chen^1,2^, Jianhai Sun^1 ,*^, Ning Xue ^1^, Wen Wang^3^, Kaisheng Jiang ^1,2^, Tianye Zhou^1 ,2^, Hao Quan ^1,2^

^1^ State Key Laboratory of Transducer Technology, Aerospace Information Research Institute, Chinese Academy of Sciences, Beijing 100194, China.

^2^ School of Electronic, Electrical and Communication Engineering, University of Chinese Academy of Sciences, Beijing 100049, China.

^3^ State Key Laboratory of Acoustics, Institute of Acoustics, Chinese Academy of Sciences, Beijing 100190, China.

Corresponding Author

* Prof. J. Sun - E-mail: [sunjh@aircas.ac.cn](mailto:sunjh@aircas.ac.cn)


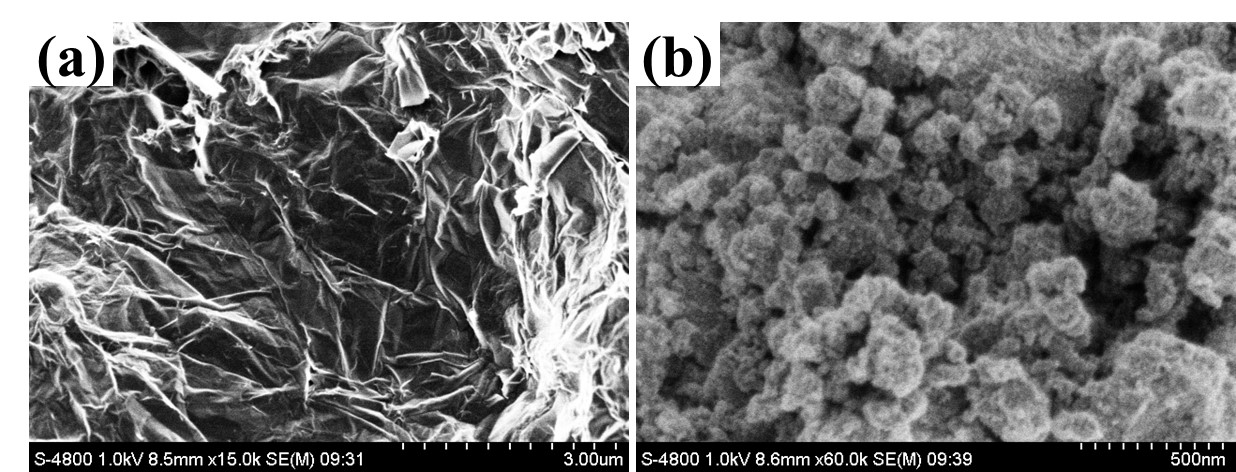


**Figure S1.** SEM patterns of rGO and Cu-SnO_2_/rGO-2.


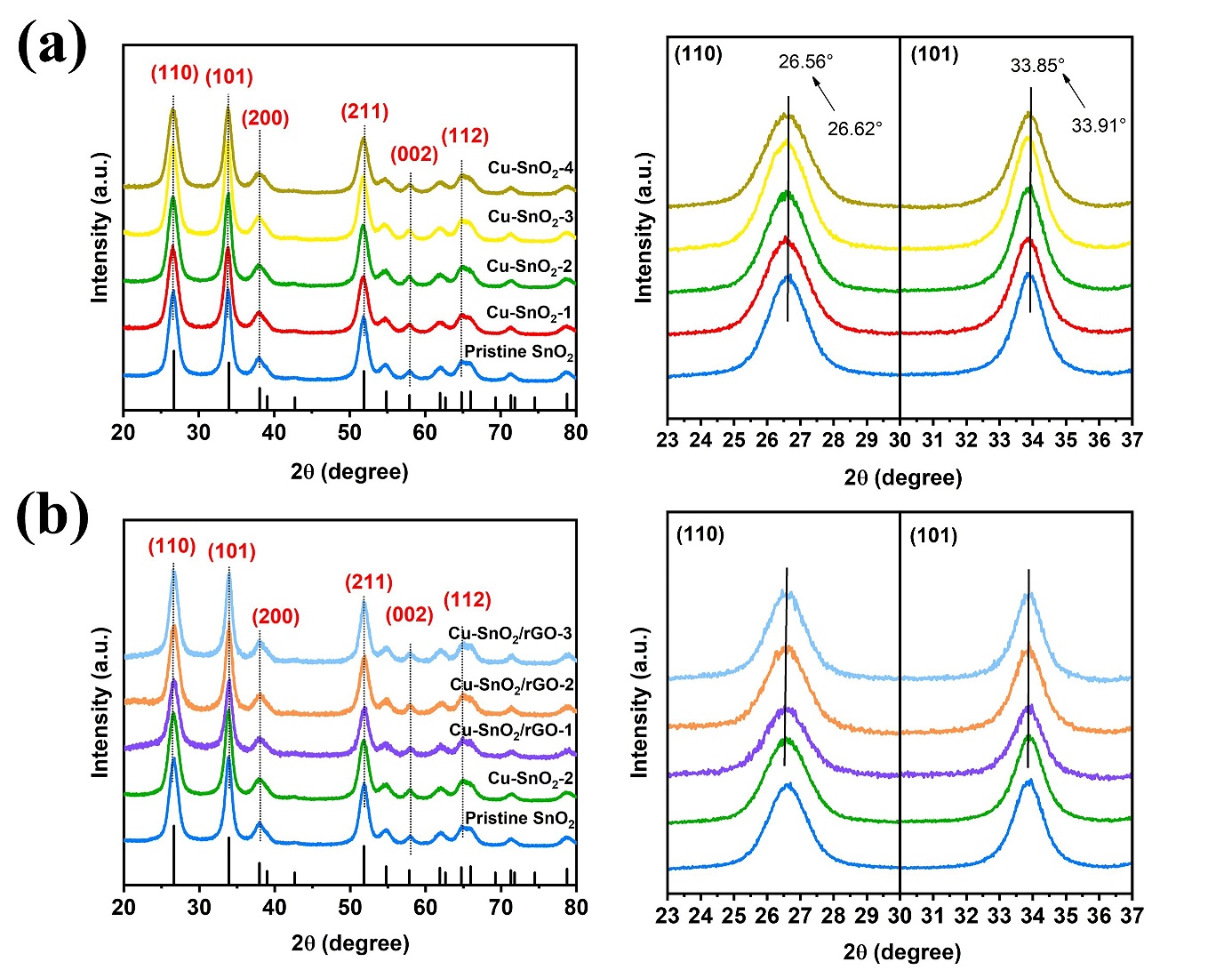


**Figure S2.** XRD patterns of samples: (a) pure SnO_2_ and Cu-SnO_2_-(1-4), (b) pure SnO_2_, Cu-SnO_2_-2, and Cu-SnO_2_/rGO-(1-3). The right side shows the corresponding enlarged views of (110) and (101) peaks.

**Table S1.** Peak position to peak area ratio for three oxygen species in O1s of pristine SnO_2_, Cu-SnO_2_-2 and Cu-SnO_2_/rGO-2.

| Materials | Absorbed oxygen (O_abs_) | | Oxygen vacancy (O_v_) | | Lattice oxygen (O_lat_) | |
| --- | --- | --- | --- | --- | --- | --- |
|  | Binding energy [eV] | Peak area [%] | Binding energy [eV] | Peak area [%] | Binding energy [eV] | Peak area [%] |
| Pristine SnO_2_ | 532.2 | 10.5 | 531.0 | 27.0 | 530.4 | 62.5 |
| Cu-SnO_2_-2 | 532.2 | 13.3 | 531.1 | 31.1 | 530.5 | 55.6 |
| Cu-SnO_2_/rGO-2 | 532.1 | 20.0 | 531.0 | 27.4 | 530.5 | 52.6 |

**Table S2.** Recent literature about H_2_S sensors based on SnO_2_.

| Sensing Materials | Temp. [℃] | Con. [ppm] | Res. ^a)^ | Res./Rec. time [s] | LOD [ppb] | Ref. |
| --- | --- | --- | --- | --- | --- | --- |
| Cu-SnO_2_/rGO | 120 | 10 | 1415.7 | 120/15 | 50 | This work |
| Cu-SnO_2_ | 180 | 100 | 25.3 | 10.1/42.4 | 10 | [1] |
| Nb_2_O_5_/SnO_2_ | 275 | 20 | 4 | 20/97 | 1 [ppm] | [2] |
| SnO_2_-CuO | 200 | 5 | 1395 | 5.27/- | 1 [ppm] | [3] |
| ZnO-SnO_2_ | 350 | 1 | 317 | ~10/450 | 100 | [4] |
| Co,N-GQDs/SnO_2_ | 260 | 100 | 37.3 | 3/13 | 50 | [5] |
| Si/SiO_2_ | 100 | 50 | 3.5 | - | 10 [ppm] | [6] |
| CNTs/SnO_2_/CuO | RT | 50 | 400 | 4/10 [min] | 10 [ppm] | [7] |
| WO_3_-coated SnO_2_ | 200 | 1 | 177 | - | 100 | [8] |
| SnO_2_@ZnO NW | 250 | 1 | 6.24 | 14/39 | 1 [ppm] | [9] |
| *a*-SnO_2_ | 250 | 1 | 2.4 | 540/- | 210 | [10] |
| LLTO-SnO_2_ | 260 | 30 | 19 | <1/60 | 500 | [11] |
| CuO-SnO_2_ | 125 | 10 | 1056 | - | - | [12] |

**Reference**

1. Zhang, S., et al. Facile fabrication of a well-ordered porous Cu-doped SnO_2_ thin film for H_2_S sensing. *ACS Appl. Mater. Interfaces* **6**, 14975-14980 (2014).
2. Mao, L. et al. Excellent long-term stable H_2_S gas sensor based on Nb_2_O_5_/SnO_2_ core-shell heterostructure nanorods. *Appl. Surf. Sci*. **602**, 154339 (2022).
3. Park, K. R. et al. Design of highly porous SnO_2_-CuO nanotubes for enhancing H_2_S gas sensor performance. *Sens. Actuators B Chem.* **302**, 127179 (2020).
4. Phuoc, P. H. et al. Comparative study on the gas-sensing performance of ZnO/SnO_2_ external and ZnO–SnO_2_ internal heterojunctions for ppb H_2_S and NO_2_ gases detection. *Sens. Actuators B Chem.* **334**, 129606 (2021).
5. Chen, T. et al. Co, N-doped GQDs/SnO_2_ mesoporous microspheres exhibit synergistically enhanced gas sensing properties for H_2_S gas detection. *J. Mater. Chem. A* **10**, 10759 (2022).
6. Bang, J. H. et al. Porous Si/SnO_2_ nanowires heterostructures for H_2_S gas sensing. *Ceram. Int.* **46**, 604-611 (2020).
7. Zhao, Y., Zhang, J., Wang, Y. & Chen, Z. A highly sensitive and room temperature CNTs/SnO_2_/CuO sensor for H_2_S gas sensing applications. *Nanoscale Res. Lett.* **15**, 1-8 (2020).
8. Hoa, T. T. N. et al. Highly selective H_2_S gas sensor based on WO_3_-coated SnO_2_ nanowires. *Mater. Today Commun.* **26**, 102094 (2021).
9. Zhu, L. et al. Hierarchical highly ordered SnO_2_ nanobowl branched ZnO nanowires for ultrasensitive and selective hydrogen sulfide gas sensing. *Microsyst. Nanoeng.* **6**, 1-13 (2020).
10. Paolucci, V. et al. Bidimensional Engineered Amorphous a-SnO_2_ Interfaces: Synthesis and Gas Sensing Response to H_2_S and Humidity. *ACS Sens.* **7**, 2058-2068 (2022).
11. Li, X. et al. Mechanism of enhanced H_2_S sensor ability based on emerging Li_0.5_La_0.5_TiO_3_-SnO_2_ core-shell structure. *Sens. Actuators B Chem.* **352**, 131054 (2022).
12. Chen, Z., Xu, Z. & Zhao, H. Flame spray pyrolysis synthesis and H_2_S sensing properties of CuO-doped SnO_2_ nanoparticles. *Proc. Combust. Inst.* **38**, 6743-6751 (2021).
